# Repair of ventricle free wall rupture after acute myocardial infarction: a case report

**DOI:** 10.1186/1757-1626-2-9099

**Published:** 2009-11-27

**Authors:** Hasan Ekim, Mustafa Tuncer, Halil Basel

**Affiliations:** 1 Yüzüncü Yil University, Department of Cardiovascular Surgery, Van, Turkey; 2 Yüzüncü Yil University, Department of Cardiology, Van, Turkey

## Abstract

**Introduction:**

Acute myocardial infarction (AMI) may culminate in sudden death by ventricular fibrillation, cardiogenic shock, and cardiac rupture. We present a case of postinfarction rupture treated by direct closure and coronary artery bypass grafting after thrombolytic therapy.

**Case report:**

A 67-year-old woman with cardiac risk factors of hypertension, diabetes mellitus, and being post-menopausal was admitted complaining of chest pain and sweating. Thrombolytic therapy with streptokinase was started due to acute myocardial infarction. But, reperfusion criteria were not achieved. Echocardiography revealed a moderate pericardial effusion with mild right chamber collapse and pericardial thrombus. Cardiac catheterization revealed totally occluded left anterior descending (LAD) and circumflex coronary arteries. She was taken to the operating-room immediately. The pericardium was opened and a large amount of blood with thrombus was removed. Her hemodynamic indices improved immediately. There was active bleeding from multiple sites with a 4 mm rupture. Cardiopulmonary bypass was established. Direct closure of rupture was carried out. Reversed autogenous saphenous vein bypass grafts were placed to the LAD and second obtuse margin coronary arteries. Postoperative recovery was uneventful and she was discharged from hospital in good condition. She remained asymptomatic during first year following the surgery.

**Conclusion:**

This case demonstrates that left ventricular free wall rupture is not always fatal and that early diagnosis and emergency surgical therapy may be successful. The combination of surgical repair with revascularization should be considered, because 80% of patients who experience LVFWR have multivessel coronary artery disease.

## Introduction

Acute myocardial infarction (AMI) may culminate in sudden death by ventricular fibrillation, cardiogenic shock, and cardiac rupture. Cardiac rupture may involve the free wall of ventricle, the interventricular septum, or papillary muscle [1]. Ventricular free wall rupture is an important but underrecognized cause of death after myocardial infarction. The real incidence is unknown [2]. Although surgical management is essential, the most appropriate surgical management remains controversial, because the experience of any surgeon or surgical group seems to be quite small [3]. However, survival without operative intervention has been described but appears to be an uncommon event [4]. We present a case of postinfarction rupture treated by direct closure and coronary artery bypass grafting after thrombolytic therapy.

## Case report

**Figure 1:**
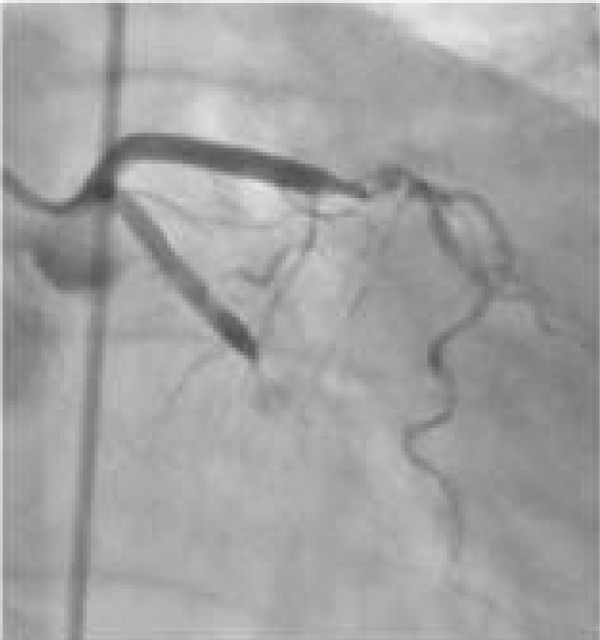
**Coronary angiography showing total occlusion of the left anterior descending and circumflex coronary arteries**.

**Figure 2:**
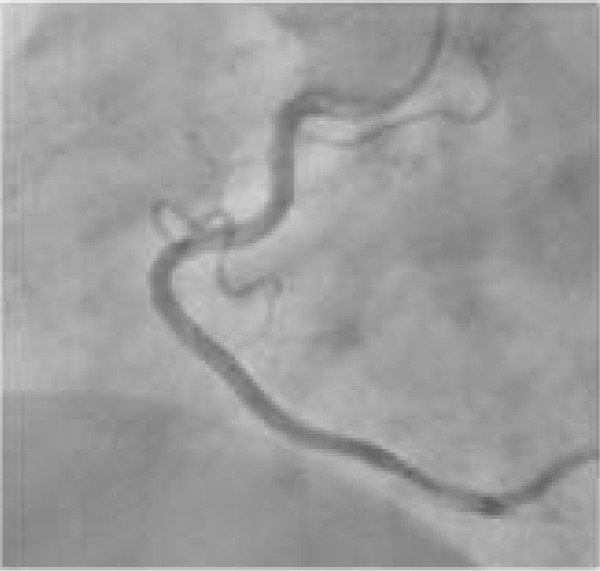
**Coronary angiography showing normal right coronary artery**.

A 67-year-old woman with cardiac risk factors of hypertension, diabetes mellitus, and being post-menopausal was admitted complaining of chest pain and sweating. Her blood pressure was 80/60 mmHg, her heart rate was 120 beats/min. Auscultation revealed no murmurs. There was ST elevation in leads D1, AVL, D2, D3, aVF, V6 and ST depression with dominant R waves in leads V1 and V2 in 12-lead electrocardiography. Oral aspirin, intravenous nitroglycerin and heparin were given. The chest radiograph was unremarkable. With the diagnosis of acute inferoposterolateral myocardial infarction, thrombolytic therapy was started. The choice of thrombolytic was streptokinase since tPA was not available and primary PCI was not possible within an acceptable time period. Reperfusion criteria were not achieved within four hours of thrombolytic therapy. Blood pressure was lowered gradually and there was dyspnea and sweating with blurred conscious. Echocardiography revealed a moderate pericardial effusion with mild right chamber collapse and pericardial thrombus. There was a turbulant flow between posteroapical left ventricular free wall and pericardial sack on color Doppler. Cardiac catheterization revealed totally occluded left anterior descending (LAD) and circumflex coronary arteries and normal right coronary artery (Figure 1, 2).

She was taken to the operating-room immediately. Median sternotomy was performed and the pericardium was opened. A large amount of blood with thrombus was removed. Her hemodynamic indices improved immediately. When the heart was lifted carefully, the area of necrosis was inspected (approximately 3 cm in diameter) in the distribution of the obtuse marginal coronary artery and was found to be extremely friable. There was active bleeding from multiple sites with a 4 mm rupture. Cardiopulmonary bypass was established by cannulation of the ascending aorta and dual stage venous cannulation through the right atrium. Moderate hypothermia was used and a left ventricular vent was inserted through the right superior pulmonary vein. Myocardial protection was achieved by means of isothermic blood cardioplegia delivered in an antegrade manner through the ascending aorta. Direct closure of rupture was carried out by two mattress sutures with Teflon felt. Reversed autogenous saphenous vein bypass grafts were placed to the LAD and second obtuse margin coronary arteries. The patient was weaned from cardiopulmonary bypass with dobutamine support. The cardiopulmonary bypass time and aortic cross-clamping time were 104 and 88 minutes, respectively. The chest was closed and drained in the usual manner.

The patient was transferred to the open heart recovery room in stable condition with inotropic support. Postoperatively, she was electively sedated and ventilated for 8 hours, during which time inotropic support was withdrawn. The patient recovered uneventfully and was discharged on the 16th postoperative day. At the 1-year follow-up visit, she was free from angina and in New York Heart Association Functional class l.

## Discussion

A cardiac rupture in patients with AMI is the second most common cause of hospital death, after pump failure. The incidence of ventricular free wall rupture after myocardial infarction has been accurately and consistently defined. It complicates approximately 4% of myocardial infarctions and accounts for 12% to 21% of deaths after infarction [5]. Four patterns of ruptures on a left ventricle have been described [5]. Type 1 exhibits an almost direct trajectory with little dissection or bloody infiltration of the myocardium. Type ll has a multicanalicular trajectory with extensive myocardial dissection and bloody infiltration. In type lll, the orifice of rupture is protected either by a thrombus or by a pericardial symphysis. Type lV is an incomplete rupture, indicating that the trajectory does not extend completely through the muscle. The terms pseudoaneurysm, simple or complex, and blow-out or ooze ruptures used in the literature describe the same pathologic condition and can easily be fitted into this classification.

In practice, it is usual that an operation is conducted within a few days after onset of AMI and low cardiac output greatly increases the surgical risk when the operation under the cardiac arrest is conducted. Moreover, repair of perforation by suture techniques raises the difficulty of sewing fragile myocardium [6]. Fortunately, as our case was active bleeding from multiple sites with a 4 mm rent, suture repair was feasible.

Sudden hypotension and bradycardia, often with cyanosis and loss of consciousness, is a frequent indicator of impending rupture. It is provoked by the entry of blood in the pericardial cavity and is often transient because the resultant small hemopericardium acts as a tampon to prevent further egress of blood [7]. The onset of rupture may be heralded by chest pain [8], which may be resistant to opiates, or by the classic features of cardiac tamponade, namely, shock with hypotension, pulsus paradoxus, elevated venous pressure, quiet heart sounds, sinus bradycardia, or frank electromechanical dissociation [5].

Intra-aortic balloon pump (IABP) support is a widely accepted treatment for ventricular septal rupture complicating AMI. However, its role in patients with left ventricular free wall rupture (LVFWR) is less clear. It is used infrequently in patients with LVFWR [9]. Older age, female sex, hypertension, and a 1st lateral or anterior wall AMI constitute traditional risk factors for LVFWR [9]. The rupture occurs typically between 1 and 7 days after the infarction. It might present suddenly with profound cardiogenic shock and cardiac tamponade [2]. There may be a predilection to first infarctions and even single vessel disease because of the paucity of collateral vessels [10].

LVFWR can be treated more conservatively by direct mattress suture buttressed with Teflon felt with or without cardiopulmonary bypass. In both techniques, the suture line must be along the nonischemic area and transmural stitches are required. Furthermore, if the sutures are placed in the necrotic myocardium, tearing could occur, particularly in the posterior wall of the left ventricle [11].

Nunez et al. [12] reported the application of a patch covering the area of infarction and anchored to normal myocardium with continuous running sutures. Because the anchoring sutures are placed only in the epicardium and shallow surface of the myocardium, myocardial damage with this technique is minimal. More recently, sutureless techniques using fibrin glue and collagen hemostats with the patch have been developed with some degree of success [13]. However, there is a caution associated with the sutureless technique. If reoperation for coronary artery bypass grafting needs to be performed, identification and exposure of the coronary artery might be difficult due to the widely and deeply piled collagen hemostats [11]. The other possible problems associated with a sutureless patch technique include the risk of recurrent rupture, pseudoaneurysm formation, and mitral valve regurgitation due to ischemic cardiomyopathy [3].

Anterior and inferoapical ruptures are easily accessed through a sternotomy, while lateral and inferobasal ruptures require mobilization of the heart and, often, unloading of the heart. The ease with which bleeding can be controlled on a beating and working heart with epicardial patching makes us wonder if the principle of avoiding cardiopulmonary bypass should not be extended to lateral and posterior rupture by directly approaching them through a left thoracotomy, especially in patients with reduced ventricular function [7]. Principles of repair of LVFWR are to stop the bleeding, anchor the repair on healthy tissue, minimize distortion of heart geometry [14], and revascularization if necessary. Association of VFWR with thrombolytic treatment has been proposed, since plasmin, activated by thrombolytic drugs, has the known effect of breaking down collagen [2]. However, the influence of thrombolysis on the incidence of rupture remains controversial, but it appears rupture occurs earlier in the course when compared with the prethrombolytic era [4].

Becker et al. [15] suggest that beginning late treatment with rt-PA is not associated with an increased risk for rupture. However, these data may not hold true for other thrombolytic agents that are not as specific as rt-PA [4]. There was a beneficial effect with a reduction in cardiac rupture rate in patients treated with streptokinase within 5 hours of symptom onset. Afterward, a significantly increased rupture rate developed in those patients who underwent delayed treatment [14]. ACC/AHA guidelines (2004) and ESC guidelines (2008) related to myocardial infarction recommend thrombolytic therapy (SKZ or tPA) in patients with true posterior myocardial infarction. Therefore, SKZ was used in our case.

## Conclusion

This case demonstrates that left ventricular free wall rupture is not always fatal and that early diagnosis and emergency surgical therapy may be successful. The combination of surgical repair with revascularization should be considered, because 80% of patients who experience LVFWR have multivessel coronary artery disease [4].

## Consent

Informed written consent was obtained for publication of the manuscript and figures.

## Competing interests

The authors declare that they have no competing interests.

## Authors' contributions

The authors performed a literature search, and wrote and approved the final manuscript.
